# Imidazolopiperazines Kill both Rings and Dormant Rings in Wild-Type and K13 Artemisinin-Resistant Plasmodium falciparum In Vitro

**DOI:** 10.1128/AAC.02235-17

**Published:** 2018-04-26

**Authors:** Laurent Dembele, Devendra Kumar Gupta, Michelle Yi-Xiu Lim, Xiaoman Ang, Jeremy J. Selva, Kesinee Chotivanich, Chea Nguon, Arjen M. Dondorp, Ghislain M. C. Bonamy, Thierry T. Diagana, Pablo Bifani

**Affiliations:** a Novartis Institute for Tropical Diseases, Singapore; b Université des Sciences, des Techniques et des Technologies de Bamako (USTTB), MRTC–DEAP–Faculty of Pharmacy, Bamako, Mali; c Mahidol-Oxford Research Unit (MORU), Faculty of Tropical Medicine, Mahidol University, Bangkok, Thailand; d Department of Clinical Tropical Medicine, Faculty of Tropical Medicine, Mahidol University, Bangkok, Thailand; e National Center for Parasitology, Entomology and Malaria Control, Phnom Penh, Cambodia; f Oxford Centre for Tropical Medicine and Global Health, Nuffield Department of Clinical Medicine, University of Oxford, Oxford, United Kingdom; g Department of Intensive Care, Academic Medical Center, University of Amsterdam, Amsterdam, The Netherlands; h Department of Microbiology and Immunology, Yong Loo Lin School of Medicine, National University of Singapore, Singapore; i Singapore Immunology Network (SIgN), A*STAR, Singapore

**Keywords:** GNF179, dormant rings, drug susceptibility, imidazolopiperazines, malaria, rings, triple therapy

## Abstract

Artemisinin (ART) resistance has spread through Southeast Asia, posing a serious threat to the control and elimination of malaria. ART resistance has been associated with mutations in the Plasmodium falciparum kelch-13 (*Pfk13*) propeller domain. Phenotypically, ART resistance is defined as delayed parasite clearance in patients due to the reduced susceptibility of early ring-stage parasites to the active metabolite of ART dihydroartemisinin (DHA). Early rings can enter a state of quiescence upon DHA exposure and resume growth in its absence. These quiescent rings are referred to as dormant rings or DHA-pretreated rings (here called dormant rings). The imidazolopiperazines (IPZ) are a novel class of antimalarial drugs that have demonstrated efficacy in early clinical trials. Here, we characterized the stage of action of the IPZ GNF179 and evaluated its activity against rings and dormant rings in wild-type and ART-resistant parasites. Unlike DHA, GNF179 does not induce dormancy. We show that GNF179 is more rapidly cidal against schizonts than against ring and trophozoite stages. However, with 12 h of exposure, the compound effectively kills rings and dormant rings of both susceptible and ART-resistant parasites within 72 h. We further demonstrate that in combination with ART, GNF179 effectively prevents recrudescence of dormant rings, including those bearing *pfk13* propeller mutations.

## INTRODUCTION

The first-line treatment for uncomplicated Plasmodium falciparum malaria recommended by the World Health Organization (WHO) is artemisinin (ART) combination therapies (ACTs) (1). Since the introduction of ATCs in the mid-1990s and implementation as first-line treatment in 2000, ACTs have had a significant impact on malaria control and the reduction of disease burden and morbidity (2, 3). In 2007, a slower parasite clearance time was first reported in artesunate-treated patients in Pailin at the Thai-Cambodian border (2). Similar reports soon followed from Cambodia, southern Vietnam, and Thailand, all the way to the Thai-Myanmar border (3–10). The exact molecular mechanism responsible for the slower parasite clearance time remains unknown, but mutations in the propeller region of the K13 gene have been shown to confer ART resistance in P. falciparum (11, 12). ART induces a dormant stage in a subfraction of P. falciparum ring-stage parasites that can cause recrudescent infection after treatment with ART monotherapy in ART-sensitive infections. Induction of dormancy might also contribute to ART resistance in ring-stage parasites (10, 13–16). Several studies have reported that following DHA treatment, there is a small percentage of developmentally arrested rings, often referred to as dormant rings (14, 17); here, DHA-pretreated rings are referred to as dormant rings and those not treated with DHA are simply rings (18). In contrast to rings, dormant rings are phenotypically resistant to ART treatment and can reenter the full life cycle after 4 days of latency (14, 19). Recently, we have shown that dormant rings are sensitive to the Plasmodium phosphatidylinositol-4-OH kinase (PI4K)-specific inhibitor KDU691, while rings are not (18).

KAF156, an imidazolopiperazine (IPZ), is a promising drug candidate showing preliminary evidence of clinical efficacy in malaria patients, including those infected with parasites bearing *Kelch13* (K13) propeller mutations (20). KAF156 displays activity against a broad range of stages of the Plasmodium life cycle, including liver and asexual blood stages as well as in gametocytes (21–23). Nevertheless, stage-specific activity within the asexual erythrocytic cycle has not been reported to date. Resistance to the IPZ KAF156 and an analogue, GNF179, has been associated with mutations in one of three *trans*-membrane transporters, *pfcarl*, *pfact*, and *pfugt* (23–25). All three of these loci have also been shown to confer resistance to other classes of experimental antimalarials, suggesting that they are not directly involved in KAF156's mechanism of action but rather are general mechanisms of drug resistance (23–25).

In this study, we aimed to carefully characterize the asexual erythrocytic stage of action of IPZ and its effects on ART-induced dormancy and phenotypic drug resistance. Using a close analog of KAF156, the IPZ GNF179 (23), we showed that IPZ (i) display the fastest cidal activity against the schizont-stage parasites, (ii) do not induce dormancy, (iii) slowly but potently kill rings and ART-induced dormant rings regardless of their *K13* genotype, and (iv) in combination with ART, IPZ but not PI4K inhibitors effectively kill wild-type and ART-resistant parasites bearing *K13* mutations.

## RESULTS

### IPZ stage of action is characterized by rapid killing of schizonts and slower but potent killing of ring-stage parasites.

**FIG 1:**
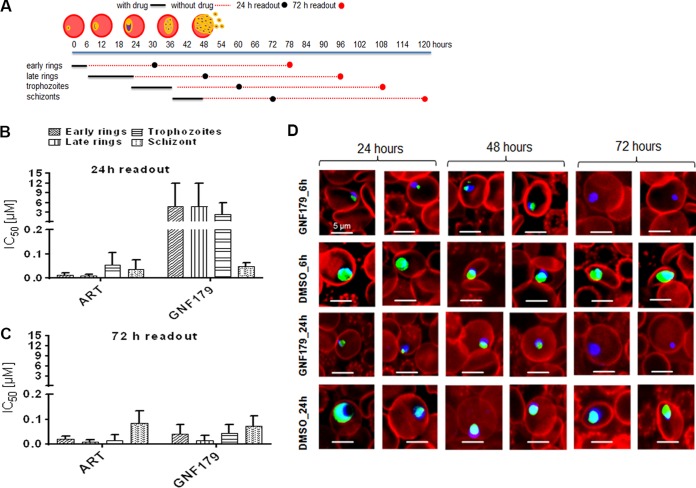
IPZ rapidly kill schizonts and slowly but potently kill ring-stage parasites without inducing dormancy. (A) Drug treatment schematic; (B) 24-h readout of 3D7 asexual blood-stage IC_50_s of GNF179 and ART using MitoTracker orange viability dye; (C) 72-h readout of 3D7 asexual blood-stage IC_50_s of GNF179 and ART using MitoTracker orange viability dye; (D) killing kinetic images of GNF179 (100 nM) after 6 and 24 h of exposure to Dd2 WT ring parasites and live parasites (green-blue) and dead parasites (blue only) using high-content imaging (HCI). Blue corresponds to DNA (DAPI stain), green corresponds to functional mitochondria (MitoTracker orange stain), and red corresponds to red blood cells (wheat germ agglutinin [WGA] conjugated to Alexa Fluor 647 stain). Drug treatment was applied on 3- to 6-h-old ring-stage parasites.

**FIG 2:**
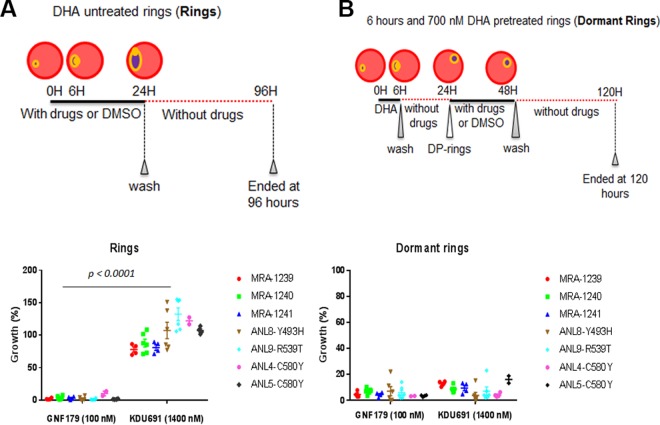
IPZ kill both ring and dormant ring parasites regardless of the K13 genotype. (A and B) IC_90_ activity against rings and dormant rings of clinical isolates of ART-resistant parasites bearing K13KDU691 (1,400 nM) (A) and GNF179 (100 nM) (B) after 72 h, detected using SYBR green. The growth controls for ring and dormant rings were DMSO-treated rings and DMSO-treated dormant rings.

**FIG 3:**
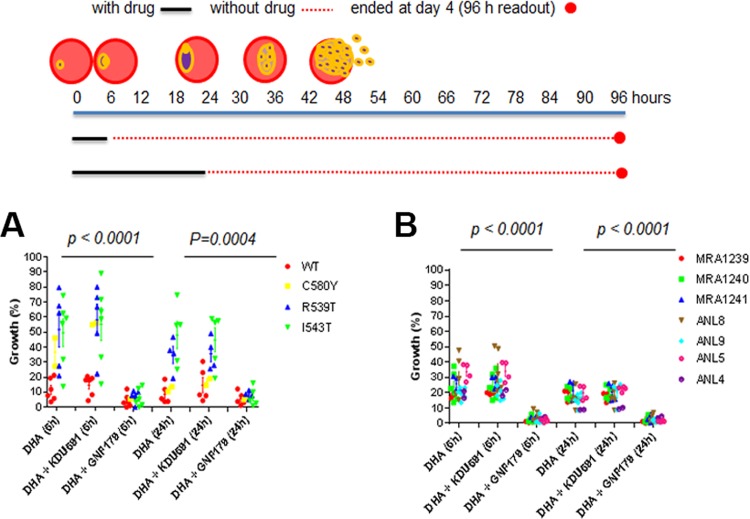
IPZ, but not PI4K inhibitors, rescue K13-mediated ART drug resistance. (A and B) Dd2 WT and Dd2 K13 mutants (C580Y, R539T, and I543T) (A) and seven clinical isolate ART-resistant parasites (B) were examined for sensitivity to DHA only or DHA (700 nM) combined either with KDU691 (1,400 nM) or GNF179 (100 nM). The growth control was DMSO-treated rings.

We have previously shown that IPZ are active against multiple stages of the Plasmodium life cycle, specifically asexual hepatic and erythrocytic stages, as well as blood and sexual stages (21, 23). In order to determine the IPZ activity against all individual asexual erythrocytic stages causing the symptomatic malaria, we conducted stage-of-action studies (schematically outlined in Fig. 1A) with the IPZ compound GNF179 (23, 24). Artemisinin (ART) is known to kill all asexual blood stages and was therefore used as a control (12, 26, 27). Briefly, parasites were tightly synchronized and treated with GNF179 in a 3-fold dilution ranging from 10 μM to 4.57 nM for the specific developmental time windows of rings (early and late), trophozoites, and schizonts (Fig. 1A, B, and C). After thorough washing, the parasites were left to grow and the viability was assessed 24 and 72 h after the end of drug treatment using the viability dye MitoTracker orange (Fig. 1). To generate 50% inhibitory concentrations (IC_50_s) for each drug and time point, IC_50_ graphs were plotted using a nonlinear regression model from quantitative growth data normalized to the dimethyl sulfoxide (DMSO) control. Cell viability/death was evaluated using MitoTracker orange, whose accumulation in the mitochondria is dependent on membrane potential, which irreversibly collapses in dead cells and cannot be positive again even after days in culture (17, 18, 28, 29). Cell viability was regularly monitored for a period of 7 days using high-content imaging (see Materials and Methods) (18). At the 24-h time point, GNF179 displayed poor activity against rings and trophozoites, while schizonts appeared to be very sensitive to GNF179 (Fig. 1B). This is in sharp contrast to the ART profile at 24 h, which was potently and rapidly cidal to rings but less active on trophozoites and schizonts (Fig. 1B). At the 72-h time point, both GNF179 and ART showed potent activity (IC_50_ of <100 nM) across all asexual blood stages (Fig. 1C). Thus, GNF179 kills rings at a lower rate than ART (Fig. 1C). While the majority of treated parasites appear viable (e.g., stained with MitoTracker dye) at 24 and 48 h, none were observed at 72 h (Fig. 1D). Collectively our results show that, similar to ART, the IPZ GNF179 displays ring-stage antimalarial activity and fast action against the schizonts. This IPZ cidal activity against rings manifests itself slowly (only after 72 h) and is observed even against very early rings (<6 h of development). We then proceeded to evaluate the activities of GNF179 against dormant rings (Fig. 1D, 2, and 3).

### IPZ kill both ring and dormant ring parasites regardless of the *K13* genotype.

It has previously been shown that after exposure to 700 nM DHA for 6 h, a small fraction of surviving or induced rings display a characteristic pyknotic-like morphology yet remain positively stained with the MitoTracker orange viability dye (17, 18). These DHA-pretreated rings (dormant rings) can resume growth a few days after ART drug removal (14). Our earlier experiments established that IPZ, similar to spiroindolones (30), do not seem to induce Plasmodium dormancy during the early ring stage (Fig. 1C and D). Rings were killed by 72 h posttreatment (Fig. 1D), when 100 nM GNF179 drug pulses were applied at 6 and 24 h (see Fig. S1 in the supplemental material). Cell viability was again regularly monitored for a period of 7 days using high-content imaging and MitoTracker orange as described above (see Materials and Methods) (17, 18, 28, 29). In addition, microscopically no parasites were identified by the less sensitive method of Giemsa staining 7 days posttreatment (Fig. S1).

The inhibitory activity of the IPZ GNF179 (23, 24) was then determined for the dormant rings. Tightly synchronized ring-stage cultures either were pretreated with 700 nM DHA to induce dormant rings or were not treated (rings) prior to a 24-h drug exposure with the IPZ GNF179. Negative (DMSO) and positive (PI4K inhibitor KDU691 [18]) controls were included. In contrast to KDU691, which only kills dormant rings (18), GNF179 significantly (*P* < 0.0001) kills both rings and dormant rings (Fig. 2A and B). This IPZ-cidal activity against the dormant rings is also observed for parasites bearing K13 mutations known to confer clinical ART drug resistance (Fig. 2A and B). Thus, IPZ can kill ring-stage parasites regardless of the *K13* genotype, developmental stage, and/or metabolism (e.g., quiescent or not).

### ART in combination with IPZ, but not PI4K inhibitors, kills wild-type and K13 ART isolates.

The dormancy of rings observed upon exposure to ART has been speculated to be an underlying physiological response mediating ART phenotypic drug resistance and prolonged clearance in patients (14, 31). Given the demonstrated cidal activity of IPZ and PI4K inhibitor KDU691 against dormant rings, we sought to determine whether these novel antimalarial compounds would be able to kill ring-stage K13-ART isolates in combination with ART. The activity of DHA alone or DHA in combination with either GNF179 (DHA-GNF179) or KDU691 (DHA-KDU691) was tested against the Dd2 WT strain, the ART-resistant Dd2 *K13* transgenic parasites (11), and clinical isolates bearing K13 mutations (Fig. 3A and B). DHA alone for 6 or 24 h was more active on the Dd2 WT strain than the *K13* transgenic lines *pfK13*-R539T and *pfK13*-I543T. The K13 transgenic parasites showed 2- to 8-fold growth increases by day four compared to Dd2 WT parasites (Fig. 3A), as expected from ART-resistant isolates and in agreement with a previous publication (18). The ART-resistant *K13* transgenic line *pfK13*-C580Y displayed moderate growth similar to that of the WT strain for 6 and 24 h of exposure, respectively (Fig. 3A). Six-hour treatment with 700 nM DHA is the condition used for selection or induction of dormant rings (18). The combination of DHA and KDU691 did not inhibit the growth of WT strains and K13 mutants at 6 and 24 h, respectively, compared to DHA alone (Fig. 3A). We have previously shown that KDU691 alone is lethal to dormant rings when drug treatment starts 24 h after exposure to 700 nM DHA for 6 h (Fig. 2B) (18). However, that does not seem to be the case when KDU691 treatment is concomitant with DHA exposure (Fig. 3A). In sharp contrast, the DHA-GNF179 drug combination proved to be highly effective against Dd2 WT and K13 mutant parasites, even after a short 6-h exposure (*P* value of <0.0001 and 0.0004 at 6 and 24 h, respectively) (Fig. 3A). Consistent with this, only DHA in combination with GNF179 potently inhibited rings from clinical isolates encoding the *K13* mutant genotype compared to inhibition by DHA or DHA plus KDU691 (*P* values of <0.0001 and <0.0001 at 6 and 24 h, respectively) (Fig. 3B). Taken together, these results show that while PI4K inhibitors may require preexposure of the rings to DHA to exert their effect on dormant rings, IPZ are immediately and potently active on rings and dormant rings concurrently exposed to DHA. Thus, ART in combination with IPZ could be a suitable partner even for ART-resistant cases.

## DISCUSSION

The emergence of ART resistance in the last decade threatens the success of global malaria control programs. While mutations in the *Kelch13* propeller domain (K13) have been identified as major determinants of ART resistance, quiescent or dormant ring-stage parasites have been associated with growth retardation, recrudescence, and phenotypic resistance to artesunate (11, 15, 32). The use of the active form of the ART prodrug dihydroartemisinin (DHA) is known to result in phenotypic resistance by inducing dormant ring formation, yet drugs withpk;1 specific activity against early and dormant rings have not been evaluated. Such drugs could be used in combination with ACT to prevent delayed response to treatment, including in cases involving a *K13* genotype. Here, we evaluated the inhibitory activity of GNF179, a close analog of the clinical candidate KAF156 (23), and the tool compound KDU691 against rings and dormant rings, alone and in combination with DHA against wild-type and K13 ART-resistant isolates. In order to discriminate dormant rings displaying a characteristic pyknotic-like appearance from dead cells, viability was determined by high-content imaging using MitoTracker orange as a viability marker (18).

Unlike DHA (14) and similar to the spiroindolones (KAE609) (30), IPZ did not induce the appearance of dormant rings. While both GNF179 and KDU691 displayed cidal activity against dormant rings, only GNF179 potently inhibited rings (Fig. 2B). Moreover, GNF179 proved to be equally active against dormant rings as well as rings even when coadministered with DHA simultaneously. We previously reported KDU691 (18) is selectively active against dormant rings but not rings. The selective dormant ring activity profile of KDU691 parallels in part the activity of mefloquine, which has also been shown to reduce the recovery of dormant rings (14) but not early rings (33). While it is possible that mefloquine is active against dormant rings, it should be noted that mefloquine has a long half-life and likely accumulates in red blood cells (RBCs), hence it could be inhibiting the development of the parasite at later stages of the life cycle (33). Mefloquine has recently been shown to inhibit protein synthesis by binding the P. falciparum 80S ribosome (34), a mechanism of action whose functions have yet to be evaluated in a dormant parasite. We have yet to determine whether the IPZ also accumulates in the RBCs. However, if inhibition resulted from accumulated compound in the cells, then the slower inhibitory activity of KAF179 observed on the early rings, late rings, and trophozoites, but faster activity on schizonts, would shift the total recovery by an additional 72 h. This is because it would target dormant rings in the recovery phase (Fig. 1 and 2). Overall, the inhibitory activity of KAF179 and mefloquine on the dormant rings should be further explored in order to better understand metabolism and phenotypic resistance in this quiescent stage of the parasite. This is particularly relevant given that mefloquine is already in use in combination therapy and has recently been associated with drug resistance (35, 36).

Surprisingly, KDU691 in combination with DHA treatment did not differ from treatment with DHA alone. This observation provides some important insights into the biology of Plasmodium dormancy, as it suggests that this physiological quiescent state is induced, and not selected for, by DHA exposure. This inference is based on the fact that KDU691 proved potent only against preexisting dormant rings and only once they have appeared posttreatment with DHA alone for 6 h (18) (Fig. 2B). In contrast, KAF179 alone and in combination potently kills rings, thereby preventing the development of dormant rings. Had DHA selected for preexisting dormant rings, DHA and KDU691 in combination should clear all parasites compared to DHA treatment alone. It is plausible that the PI4K, the target of KDU691, is not expressed in the early rings but that it is essential in the dormant ring stages. The activity of GNF179 was further evaluated against each asexual erythrocytic stage of the parasite, where it proved to be fast acting against the schizont stage and slowly kill during ring and trophozoite stages. This finding may facilitate further studies establishing IPZ molecular mechanisms of action.

Combination therapies such as ACTs are essential in reducing the emergence of drug resistance. Nevertheless, while ART resistance has now been widely reported in Southeast Asia and China (37), it has not been reported as of yet in Africa (38), South America, or South Asia (39). In the absence of resistance to long-half-life ACT partner drugs, such as lumefantrine, amodiaquine, mefloquine, and piperaquine, ACT clinical efficacy for treatment of falciparum malaria is largely preserved. However, ART resistance facilitates selection of partner drug resistance. This has been observed in Cambodia and at the Thai-Myanmar border, where emergence of ART resistance was followed by a fast increase in piperaquine and mefloquine resistance, causing high ACT treatment failure (40–43). Recent reports from Vietnam indicate treatment failure was associated with mutations in the K13 target gene and the *Pfplasmepsin2* (*pfpm2*) gene associated with a partner drug, causing piperaquine resistance (44). More recently, a strain resistant to ACT (artemisinin and piperaquine) spread from western Cambodia to southern Vietnam through northeastern Thailand and southern Laos (45). Introduction of ART and partner drug resistance spread from Southeast Asia, or as *de novo* emergence, would be disastrous for the African continent. Although current reports have not convincingly shown that ART resistance has been established in any sub-Saharan African country, this might unfortunately only be a matter of time (39). These early signs of treatment failures to ACT may be an indication that the time has come to consider new combinations of ART with novel mechanism-of-action compounds or triple therapy. We have previously shown that the spiroindolone KAE609 is fast acting at low-nanomolar concentrations but that in contrast to ART, it does not induce dormancy and has a longer half-life (23 h) capable of preventing recrudescence, including that from dormant rings (2, 9, 30, 46, 47). Our study shows that IPZ with a mean half-life of 44.1 ± 8.9 h in humans (20) potently inhibited both early and dormant rings of wild-type as well as K13 ART-resistant parasites without inducing dormancy. The data reported here suggest that this novel class of antimalarial is a suitable drug partner for ACT combination therapy to prevent the recrudescence of dormant rings in K13 ART-resistant isolates.

## MATERIALS AND METHODS

### Antimalarial drugs.

Compounds KDU691 and GNF179 were synthesized in-house (48, 49). All other compounds used in the study were obtained from Sigma-Aldrich (St. Louis, MO, USA).

### Parasites.

P. falciparum field clinical isolates ANL4-C580Y, ANL5-C580Y, ANL8-Y493H, and ANL9-R539T, encoding *K13* mutations, were obtained from Mahidol University, Bangkok, Thailand (50). Samples were collected under approved ethical guidelines of the Oxford Tropical Research Ethics Committee (OXTREC 562-15) and the Faculty of Tropical Medicine, Mahidol University, Bangkok, Thailand (MUTM 2015-019-01). All sample collection was performed in accordance with the relevant guidelines and regulations (OXTREC 562-15 and MUTM 2015-019-01).

MRA-1239, MRA-1240, MRA-1241, and the corresponding ring-stage survival assay (RSA) data were obtained from BEI Resources. Strains MRA-1239, MRA-1240, and MRA-1241 were obtained from The Malaria Research and Reference Reagent Resource Centre (MR4), USA. Strain MRA-1239 (IPC 5188; susceptible in an RSA from 0 to 3 h [RSA_0–3_]) was originally isolated in 2011 from the blood of a human patient with malaria in Ratanakiri province, northeastern Cambodia. When exposed to dihydroartemisinin, the strain gave an RSA_0–3_ value of 0.1%. Strain MRA-1240 (IPC5202; resistant in RSA_0–3_) was originally isolated in 2011 from a human patient with malaria in Battambang province, western Cambodia. When exposed to dihydroartemisinin, it gave an RSA_0–3_ value of 88.2%. Strain MRA-1241 (IPC4912; resistant in RSA_0–3_) was originally isolated in 2011 from the blood of a human patient with malaria in Mondulkiri province, southeastern Cambodia. When exposed to dihydroartemisinin, it gave an RSA_0–3_ value of 49.3%.

P. falciparum laboratory-adapted parental strain Dd2 (a clone of W2MEF) and ART-resistant transgenic lines with mutations (R539T, I543T, and C580Y) in the *K13* gene were a kind gift from the David Fidock laboratory, Columbia University, New York, NY (51).

### Parasite cultures.

All clinical isolates and laboratory-adapted strains of P. falciparum were cultured using standard RPMI 1640-HEPES (Gibco Life Technologies, Singapore) medium supplemented with 0.5% AlbuMAX and 4% RBCs. RBCs used in this study were obtained from Innovative Research, USA. Parasites were synchronized with 5% d-sorbitol at each cycle and prior to drug exposure in each experiment (52).

### GNF179 stage of action.

A drug-sensitive P. falciparum 3D7 culture was synchronized for a week prior to the start of the experiments. The asynchronous 3D7 culture was first synchronized using MACS purification (Miltenyi Biotec) to obtain pure schizonts. The second synchronization with 5% sorbitol at the ring stage was carried out 12 h preceding the next MACS purification. The alternating sorbitol and MACS purification program was repeated for three life cycles with a final purification using the MACS column for mature schizonts. To facilitate reinvasion of merozoites into red blood cells, the culture was left in a shaking incubator at 40 rpm for 4 h after MACS purification. The repeated synchronization of cultures gave a tight 4- to 6-h window of parasite growth. The aim of the experiment was to investigate the effect GNF179 and ART control had on early rings (0 to 6 h postinvasion [p.i.]), rings (7 to 24 h p.i.), trophozoites (25 to 38 h p.i.), and schizonts (39 to 48 h p.i.).

A 96-well master plate containing ART and GNF179 was prepared, with the highest concentration at 10 μM and 3-fold serial dilutions to yield eight concentration points. A final working concentration of 0.1% DMSO was used as the negative control, and 10 μM ART was used as the positive control. Each well was spotted with 200 nl of compound using a Mosquito nanoliter dispenser (Cambridge, UK). At different hours postinvasion of the red blood cells, early rings (0 h p.i.), late rings (6 h p.i.), trophozoites (24 h p.i.), and schizonts (36 h p.i.) in 200 μl of P. falciparum 3D7 culture at the respective intraerythrocytic stage were seeded at 0.5% parasitemia and 4.0% hematocrit manually into the compound-spotted plates. The plates were incubated in an incubator with a reduced oxygen environment and 5% CO_2_ at 37°C. Giemsa-stained slides were prepared prior to seeding to ensure the culture was at the correct intraerythrocytic stage.

The culture at the respective intraerythrocytic stage was incubated with the compounds for a number of hours before the compounds were washed off extensively, twice with 1× phosphate-buffered saline (PBS) and once with complete RPMI medium. The incubation times were the following: early rings, 6 h; late rings, 18 h; trophozoites, 12 h; and schizonts, 12 h. Upon the removal of the compounds, the stage-specific parasites were cultured in complete RPMI medium before being assessed with MitoTracker orange for their IC_50_ determinations at 24 h and 72 h (see below).

### HCI.

HCI was carried out on an Opera (PerkinElmer) high-content screening system, and the fluorescent dye MitoTracker orange was used to monitor parasite growth. The HCI readout measures only the absolute live parasite count per field base on MitoTracker orange staining only and not as a percentage of parasitemia. GraphPad Prism 7 software was used to make the analysis and generate the graphs. For detection with a final concentration of 250 nM MitoTracker orange, 170 μl of medium was removed from each of the 96-well plates, and an equal volume of MitoTracker orange in PBS was added to the remaining 30 μl of infected culture. The plates were incubated for 24 h at 37°C before being read on the Opera high-content imaging system (PerkinElmer). Each well was imaged bottom-up for 25 fields using a 561-nm laser.

The wells were imaged for live cells stained with MitoTracker orange. Parasites were counted in a total of 20 fields per well. The number of live parasites was quantified using a custom Acapalla script and algorithms for high-content imaging. Quantitative data were normalized to the control (DMSO), and IC_50_ graphs were plotted using a nonlinear regression model. Bar graphs were plotted using GraphPad Prism7.

### GNF179 rings killing kinetic.

Sorbitol (52)-synchronized rings (1% parasitemia) were directly exposed for 6 and 24 h to 100 nM GNF179 and incubated at 37°C in 5% CO_2_. After the 6- and 24-h treatments, drug was removed by three consecutive washes and parasite growth was monitored for 24 h, 48 h, and 72 h as shown in Fig. 1D. MitoTracker-positive and -negative parasites were evaluated as follows. Parasite nuclei (blue) were stained with 1 μg/ml of 4′,6-diamidino-2-phenylindole (DAPI; Sigma), and mitochondria (green) were stained with a 2 μM final concentration of MitoTracker orange (dissolved in culture media together with DAPI) for 2 h at 37°C in 5% CO_2_. After 2 h of staining, medium was removed and 1/500 diluted wheat germ agglutinin (WGA), conjugated with Alexa Fluor 647 (1 mg/ml stock concentration), was used to stain the red blood cells in 1× PBS for 10 min and then washed twice in 1× PBS. Images were taken with an Opera (PerkinElmer) high-content screening system at 60× magnification (Fig. 1D).

### Ring and dormant ring drug susceptibility assessment.

All parasites were first synchronized at ring stage with sorbitol (52). In single-drug treatment (Fig. 2), equal levels of parasitemia adjusted to 0.1% were used for both rings and dormant rings. Dormant rings were induced as described above and previously reported (14). Synchronized ring parasites were either exposed directly to compound pulses at concentrations corresponding to their respective 72-h SYBR green assay IC_90_ values (see Fig. S2 in the supplemental material) for 24 h (for rings) or pretreated with DHA (700 nM) for 6 h, washed thrice to remove the DHA, and then exposed 18 h later to the compound treatment for 24 h (for dormant rings). In combination treatment (Fig. 3), 700 nM DHA, previously reported to induce dormancy or ring-stage survival, was used (15, 16). DHA alone or DHA plus one of the partner drugs (GNF179 or KDU691) was used. Synchronized rings (0.1% parasitemia) were directly exposed for 6 and 24 h to DHA plus one partner drug or to DHA alone to determine 72-h SYBR green assay IC_90_ values. A DMSO-treated sample was used as a control. Treatment was done with GNF179 (100 nM), KDU691 (1,400 nM), and DHA (700 nM). Drug- and DMSO-treated parasites were stained with MitoTracker orange (250 nM final concentration in culture medium for 24 h at 37°C in 5% CO_2_) and analyzed with a PerkinElmer Opera HCI system. The uptake of MitoTracker orange is dependent on the negative mitochondrial membrane potential and indicative of cell viability (17, 53). For high-content imaging, cultures containing MitoTracker orange were adjusted to 2% hematocrit in Greiner PS microplates (black cell culture, 96-well, F-bottom, μClear plate format; product code 655090). Following 6 and 24 h of treatment, drugs were removed from culture medium by three consecutive washes at the end of 6 and 24 h of exposure using 1× PBS, and parasite growth was assessed by day four. All drug-treated conditions were normalized to corresponding DMSO-treated conditions. All data were obtained from three or more independent biological experiments with technical duplicates or triplicates (means ± standard errors of the means). Statistical analyses of data were done using a Mann-Whitney U test.

## Supplementary Material

Supplemental material

## References

[1] WHO. 2015 World malaria report. World Health Organization, Geneva, Switzerland.

[2] Dondorp AM, Nosten F, Yi P, Das D, Phyo AP, Tarning J, Lwin KM, Ariey F, Hanpithakpong W, Lee SJ, Ringwald P, Silamut K, Imwong M, Chotivanich K, Lim P, Herdman T, An SS, Yeung S, Singhasivanon P, Day NP, Lindegardh N, Socheat D, White NJ 2009 Artemisinin resistance in Plasmodium falciparum malaria. N Engl J Med 361:455–467. doi:10.1056/NEJMoa0808859. 19641202 PMC3495232

[3] White NJ 2008 Qinghaosu (artemisinin): the price of success. Science 320:330–334. doi:10.1126/science.1155165. 18420924

[4] Phyo AP, Ashley EA, Anderson TJ, Bozdech Z, Carrara VI, Sriprawat K, Nair S, White MM, Dziekan J, Ling C, Proux S, Konghahong K, Jeeyapant A, Woodrow CJ, Imwong M, McGready R, Lwin KM, Day NP, White NJ, Nosten F 2016 Declining efficacy of artemisinin combination therapy against P. falciparum malaria on the Thai-Myanmar border (2003-2013): the role of parasite genetic factors. Clin Infect Dis 63:784–791. doi:10.1093/cid/ciw388. 27313266 PMC4996140

[5] Leang R, Taylor WR, Bouth DM, Song L, Tarning J, Char MC, Kim S, Witkowski B, Duru V, Domergue A, Khim N, Ringwald P, Menard D 2015 Evidence of Plasmodium falciparum malaria multidrug resistance to artemisinin and piperaquine in western Cambodia: dihydroartemisinin-piperaquine open-label multicenter clinical assessment. Antimicrob Agents Chemother 59:4719–4726. doi:10.1128/AAC.00835-15. 26014949 PMC4505193

[6] Farnert A, Ursing J, Tolfvenstam T, Rono J, Karlsson L, Sparrelid E, Lindegardh N 2012 Artemether-lumefantrine treatment failure despite adequate lumefantrine day 7 concentration in a traveller with Plasmodium falciparum malaria after returning from Tanzania. Malar J 11:176. doi:10.1186/1475-2875-11-176. 22632033 PMC3416680

[7] Sisowath C, Ferreira PE, Bustamante LY, Dahlstrom S, Martensson A, Bjorkman A, Krishna S, Gil JP 2007 The role of pfmdr1 in Plasmodium falciparum tolerance to artemether-lumefantrine in Africa. Trop Med Int Health 12:736–742. doi:10.1111/j.1365-3156.2007.01843.x. 17550470

[8] Phyo AP, Nkhoma S, Stepniewska K, Ashley EA, Nair S, McGready R, ler Moo C, Al-Saai S, Dondorp AM, Lwin KM, Singhasivanon P, Day NP, White NJ, Anderson TJ, Nosten F 2012 Emergence of artemisinin-resistant malaria on the western border of Thailand: a longitudinal study. Lancet 379:1960–1966. doi:10.1016/S0140-6736(12)60484-X. 22484134 PMC3525980

[9] Hien TT, Thuy-Nhien NT, Phu NH, Boni MF, Thanh NV, Nha-Ca NT, Thai le H, Thai CQ, Toi PV, Thuan PD, Long le T, Dong le T, Merson TL, Dolecek C, Stepniewska K, Ringwald P, White NJ, Farrar J, Wolbers M 2012 In vivo susceptibility of Plasmodium falciparum to artesunate in Binh Phuoc Province, Vietnam. Malar J 11:355. doi:10.1186/1475-2875-11-355. 23101492 PMC3504531

[10] Ashley EA, Dhorda M, Fairhurst RM, Amaratunga C, Lim P, Suon S, Sreng S, Anderson JM, Mao S, Sam B, Sopha C, Chuor CM, Nguon C, Sovannaroth S, Pukrittayakamee S, Jittamala P, Chotivanich K, Chutasmit K, Suchatsoonthorn C, Runcharoen R, Hien TT, Thuy-Nhien NT, Thanh NV, Phu NH, Htut Y, Han KT, Aye KH, Mokuolu OA, Olaosebikan RR, Folaranmi OO, Mayxay M, Khanthavong M, Hongvanthong B, Newton PN, Onyamboko MA, Fanello CI, Tshefu AK, Mishra N, Valecha N, Phyo AP, Nosten F, Yi P, Tripura R, Borrmann S, Bashraheil M, Peshu J, Faiz MA, Ghose A, Hossain MA, Samad R, 2014 Spread of artemisinin resistance in Plasmodium falciparum malaria. N Engl J Med 371:411–423. doi:10.1056/NEJMoa1314981. 25075834 PMC4143591

[11] Straimer J, Gnadig NF, Witkowski B, Amaratunga C, Duru V, Ramadani AP, Dacheux M, Khim N, Zhang L, Lam S, Gregory PD, Urnov FD, Mercereau-Puijalon O, Benoit-Vical F, Fairhurst RM, Menard D, Fidock DA 2015 Drug resistance. K13-propeller mutations confer artemisinin resistance in Plasmodium falciparum clinical isolates. Science 347:428–431. 25502314 10.1126/science.1260867 PMC4349400

[12] Tilley L, Straimer J, Gnadig NF, Ralph SA, Fidock DA 2016 Artemisinin action and resistance in Plasmodium falciparum. Trends Parasitol 32:682–696. doi:10.1016/j.pt.2016.05.010. 27289273 PMC5007624

[13] Menard S, Ben Haddou T, Ramadani AP, Ariey F, Iriart X, Beghain J, Bouchier C, Witkowski B, Berry A, Mercereau-Puijalon O, Benoit-Vical F 2015 Induction of multidrug tolerance in Plasmodium falciparum by extended artemisinin pressure. Emerg Infect Dis 21:1733–1741. doi:10.3201/eid2110.150682. 26401601 PMC4593447

[14] Teuscher F, Gatton ML, Chen N, Peters J, Kyle DE, Cheng Q 2010 Artemisinin-induced dormancy in plasmodium falciparum: duration, recovery rates, and implications in treatment failure. J Infect Dis 202:1362–1368. doi:10.1086/656476. 20863228 PMC2949454

[15] Witkowski B, Amaratunga C, Khim N, Sreng S, Chim P, Kim S, Lim P, Mao S, Sopha C, Sam B, Anderson JM, Duong S, Chuor CM, Taylor WR, Suon S, Mercereau-Puijalon O, Fairhurst RM, Menard D 2013 Novel phenotypic assays for the detection of artemisinin-resistant Plasmodium falciparum malaria in Cambodia: in-vitro and ex-vivo drug-response studies. Lancet Infect Dis 13:1043–1049. doi:10.1016/S1473-3099(13)70252-4. 24035558 PMC5015432

[16] Witkowski B, Lelievre J, Barragan MJ, Laurent V, Su XZ, Berry A, Benoit-Vical F 2010 Increased tolerance to artemisinin in Plasmodium falciparum is mediated by a quiescence mechanism. Antimicrob Agents Chemother 54:1872–1877. doi:10.1128/AAC.01636-09. 20160056 PMC2863624

[17] Peatey CL, Chavchich M, Chen N, Gresty KJ, Gray KA, Gatton ML, Waters NC, Cheng Q 2015 A small subset of artemisinin induced dormant P. falciparum parasites maintain mitochondrial membrane potential and resume growth in vitro. J Infect Dis 212:426–434. doi:10.1093/infdis/jiv048. 25635122

[18] Dembele L, Ang X, Chavchich M, Bonamy GMC, Selva JJ, Lim MY, Bodenreider C, Yeung BKS, Nosten F, Russell BM, Edstein MD, Straimer J, Fidock DA, Diagana TT, Bifani P 2017 The Plasmodium PI(4)K inhibitor KDU691 selectively inhibits dihydroartemisinin-pretreated Plasmodium falciparum ring-stage parasites. Sci Rep 7:2325. doi:10.1038/s41598-017-02440-6. 28539634 PMC5443816

[19] Teuscher F, Chen N, Kyle DE, Gatton ML, Cheng Q 2012 Phenotypic changes in artemisinin-resistant Plasmodium falciparum lines in vitro: evidence for decreased sensitivity to dormancy and growth inhibition. Antimicrob Agents Chemother 56:428–431. doi:10.1128/AAC.05456-11. 21986828 PMC3256014

[20] White NJ, Duong TT, Uthaisin C, Nosten F, Phyo AP, Hanboonkunupakarn B, Pukrittayakamee S, Jittamala P, Chuthasmit K, Cheung MS, Feng Y, Li R, Magnusson B, Sultan M, Wieser D, Xun X, Zhao R, Diagana TT, Pertel P, Leong FJ 2016 Antimalarial activity of KAF156 in falciparum and vivax malaria. N Engl J Med 375:1152–1160. doi:10.1056/NEJMoa1602250. 27653565 PMC5142602

[21] Kuhen KL, Chatterjee AK, Rottmann M, Gagaring K, Borboa R, Buenviaje J, Chen Z, Francek C, Wu T, Nagle A, Barnes SW, Plouffe D, Lee MC, Fidock DA, Graumans W, van de Vegte-Bolmer M, van Gemert GJ, Wirjanata G, Sebayang B, Marfurt J, Russell B, Suwanarusk R, Price RN, Nosten F, Tungtaeng A, Gettayacamin M, Sattabongkot J, Taylor J, Walker JR, Tully D, Patra KP, Flannery EL, Vinetz JM, Renia L, Sauerwein RW, Winzeler EA, Glynne RJ, Diagana TT 2014 KAF156 is an antimalarial clinical candidate with potential for use in prophylaxis, treatment, and prevention of disease transmission. Antimicrob Agents Chemother 58:5060–5067. doi:10.1128/AAC.02727-13. 24913172 PMC4135840

[22] Leong FJ, Zhao R, Zeng S, Magnusson B, Diagana TT, Pertel P 2014 A first-in-human randomized, double-blind, placebo-controlled, single- and multiple-ascending oral dose study of novel imidazolopiperazine KAF156 to assess its safety, tolerability, and pharmacokinetics in healthy adult volunteers. Antimicrob Agents Chemother 58:6437–6443. doi:10.1128/AAC.03478-14. 25136017 PMC4249437

[23] Meister S, Plouffe DM, Kuhen KL, Bonamy GM, Wu T, Barnes SW, Bopp SE, Borboa R, Bright AT, Che J, Cohen S, Dharia NV, Gagaring K, Gettayacamin M, Gordon P, Groessl T, Kato N, Lee MC, McNamara CW, Fidock DA, Nagle A, Nam TG, Richmond W, Roland J, Rottmann M, Zhou B, Froissard P, Glynne RJ, Mazier D, Sattabongkot J, Schultz PG, Tuntland T, Walker JR, Zhou Y, Chatterjee A, Diagana TT, Winzeler EA 2011 Imaging of Plasmodium liver stages to drive next-generation antimalarial drug discovery. Science 334:1372–1377. doi:10.1126/science.1211936. 22096101 PMC3473092

[24] Lim MY, LaMonte G, Lee MC, Reimer C, Tan BH, Corey V, Tjahjadi BF, Chua A, Nachon M, Wintjens R, Gedeck P, Malleret B, Renia L, Bonamy GM, Ho PC, Yeung BK, Chow ED, Lim L, Fidock DA, Diagana TT, Winzeler EA, Bifani P 2016 UDP-galactose and acetyl-CoA transporters as Plasmodium multidrug resistance genes. Nat Microbiol 19:16166. doi:10.1038/nmicrobiol.2016.166. PMC5575994 27642791

[25] LaMonte G, Lim MY, Wree M, Reimer C, Nachon M, Corey V, Gedeck P, Plouffe D, Du A, Figueroa N, Yeung B, Bifani P, Winzeler EA 2016 Mutations in the Plasmodium falciparum cyclic amine resistance locus (PfCARL) confer multidrug resistance. mBio 7:e00696-16. 27381290 10.1128/mBio.00696-16 PMC4958248

[26] Xie SC, Dogovski C, Hanssen E, Chiu F, Yang T, Crespo MP, Stafford C, Batinovic S, Teguh S, Charman S, Klonis N, Tilley L 2016 Haemoglobin degradation underpins the sensitivity of early ring stage Plasmodium falciparum to artemisinins. J Cell Sci 129:406–416. doi:10.1242/jcs.178830. 26675237 PMC4732288

[27] Klonis N, Xie SC, McCaw JM, Crespo-Ortiz MP, Zaloumis SG, Simpson JA, Tilley L 2013 Altered temporal response of malaria parasites determines differential sensitivity to artemisinin. Proc Natl Acad Sci U S A 110:5157–5162. doi:10.1073/pnas.1217452110. 23431146 PMC3612604

[28] Fernandez-Gomez FJ, Galindo MF, Gomez-Lazaro M, Yuste VJ, Comella JX, Aguirre N, Jordan J 2005 Malonate induces cell death via mitochondrial potential collapse and delayed swelling through an ROS-dependent pathway. Br J Pharmacol 144:528–537. doi:10.1038/sj.bjp.0706069. 15655518 PMC1576031

[29] Scorrano L, Petronilli V, Di Lisa F, Bernardi P 1999 Commitment to apoptosis by GD3 ganglioside depends on opening of the mitochondrial permeability transition pore. J Biol Chem 274:22581–22585. doi:10.1074/jbc.274.32.22581. 10428836

[30] Chavchich M, Van Breda K, Rowcliffe K, Diagana TT, Edstein MD 2016 The siroindolone KAE609 does not induce dormant ring stages in Plasmodium falciparum parasites. Antimicrob Agents Chemother 60:5167–5174. doi:10.1128/AAC.02838-15. 27297484 PMC4997877

[31] LaCrue AN, Scheel M, Kennedy K, Kumar N, Kyle DE 2011 Effects of artesunate on parasite recrudescence and dormancy in the rodent malaria model Plasmodium vinckei. PLoS One 6:e26689. doi:10.1371/journal.pone.0026689. 22039533 PMC3200358

[32] Witkowski B, Khim N, Chim P, Kim S, Ke S, Kloeung N, Chy S, Duong S, Leang R, Ringwald P, Dondorp AM, Tripura R, Benoit-Vical F, Berry A, Gorgette O, Ariey F, Barale JC, Mercereau-Puijalon O, Menard D 2013 Reduced artemisinin susceptibility of Plasmodium falciparum ring stages in western Cambodia. Antimicrob Agents Chemother 57:914–923. doi:10.1128/AAC.01868-12. 23208708 PMC3553720

[33] Wilson DW, Langer C, Goodman CD, McFadden GI, Beeson JG 2013 Defining the timing of action of antimalarial drugs against Plasmodium falciparum. Antimicrob Agents Chemother 57:1455–1467. doi:10.1128/AAC.01881-12. 23318799 PMC3591904

[34] Wong W, Bai XC, Sleebs BE, Triglia T, Brown A, Thompson JK, Jackson KE, Hanssen E, Marapana DS, Fernandez IS, Ralph SA, Cowman AF, Scheres SHW, Baum J 2017 Mefloquine targets the Plasmodium falciparum 80S ribosome to inhibit protein synthesis. Nat Microbiol 2:17031. doi:10.1038/nmicrobiol.2017.31. 28288098 PMC5439513

[35] Looareesuwan S, Kyle DE, Viravan C, Vanijanonta S, Wilairatana P, Charoenlarp P, Canfield CJ, Webster HK 1992 Treatment of patients with recrudescent falciparum malaria with a sequential combination of artesunate and mefloquine. Am J Trop Med Hyg 47:794–799. doi:10.4269/ajtmh.1992.47.794. 1471736

[36] Nosten F, van Vugt M, Price R, Luxemburger C, Thway KL, Brockman A, McGready R, ter Kuile F, Looareesuwan S, White NJ 2000 Effects of artesunate-mefloquine combination on incidence of Plasmodium falciparum malaria and mefloquine resistance in western Thailand: a prospective study. Lancet 356:297–302. doi:10.1016/S0140-6736(00)02505-8. 11071185

[37] Menard D, Khim N, Beghain J, Adegnika AA, Shafiul-Alam M, Amodu O, Rahim-Awab G, Barnadas C, Berry A, Boum Y, Bustos MD, Cao J, Chen JH, Collet L, Cui L, Thakur GD, Dieye A, Djalle D, Dorkenoo MA, Eboumbou-Moukoko CE, Espino FE, Fandeur T, Ferreira-da-Cruz MF, Fola AA, Fuehrer HP, Hassan AM, Herrera S, Hongvanthong B, Houze S, Ibrahim ML, Jahirul-Karim M, Jiang L, Kano S, Ali-Khan W, Khanthavong M, Kremsner PG, Lacerda M, Leang R, Leelawong M, Li M, Lin K, Mazarati JB, Menard S, Morlais I, Muhindo-Mavoko H, Musset L, Na-Bangchang K, Nambozi M, Niare K, Noedl H, 2016 A worldwide map of Plasmodium falciparum K13-propeller polymorphisms. N Engl J Med 374:2453–2464. doi:10.1056/NEJMoa1513137. 27332904 PMC4955562

[38] Sutherland CJ, Lansdell P, Sanders M, Muwanguzi J, van Schalkwyk DA, Kaur H, Nolder D, Tucker J, Bennett HM, Otto TD, Berriman M, Patel TA, Lynn R, Gkrania-Klotsas E, Chiodini PL 2017 pfk13-independent treatment failure in four imported cases of Plasmodium falciparum malaria treated with artemether-lumefantrine in the United Kingdom. Antimicrob Agents Chemother 61:e02382-16. doi:10.1128/AAC.02382-16. 28137810 PMC5328508

[39] Daily JP 2016 K13-propeller mutations and malaria resistance. N Engl J Med 374:2492–2493. doi:10.1056/NEJMe1604520. 27332909

[40] Spring MD, Lin JT, Manning JE, Vanachayangkul P, Somethy S, Bun R, Se Y, Chann S, Ittiverakul M, Sia-Ngam P, Kuntawunginn W, Arsanok M, Buathong N, Chaorattanakawee S, Gosi P, Ta-aksorn W, Chanarat N, Sundrakes S, Kong N, Heng TK, Nou S, Teja-Isavadharm P, Pichyangkul S, Phann ST, Balasubramanian S, Juliano JJ, Meshnick SR, Chour CM, Prom S, Lanteri CA, Lon C, Saunders DL 2015 Dihydroartemisinin-piperaquine failure associated with a triple mutant including kelch13 C580Y in Cambodia: an observational cohort study. Lancet Infect Dis 15:683–691. doi:10.1016/S1473-3099(15)70049-6. 25877962

[41] Saunders DL, Vanachayangkul P, Lon C, U.S. Army Military Malaria Research Program, National Center for Parasitology, Entomology, and Malaria Control (CNM), Royal Cambodian Armed Forces. 2014 Dihydroartemisinin-piperaquine failure in Cambodia. N Engl J Med 371:484–485. doi:10.1056/NEJMc1403007. 25075853

[42] Rogers WO, Sem R, Tero T, Chim P, Lim P, Muth S, Socheat D, Ariey F, Wongsrichanalai C 2009 Failure of artesunate-mefloquine combination therapy for uncomplicated Plasmodium falciparum malaria in southern Cambodia. Malar J 8:10. doi:10.1186/1475-2875-8-10. 19138388 PMC2628668

[43] Amaratunga C, Lim P, Suon S, Sreng S, Mao S, Sopha C, Sam B, Dek D, Try V, Amato R, Blessborn D, Song L, Tullo GS, Fay MP, Anderson JM, Tarning J, Fairhurst RM 2016 Dihydroartemisinin-piperaquine resistance in Plasmodium falciparum malaria in Cambodia: a multisite prospective cohort study. Lancet Infect Dis 16:357–365. doi:10.1016/S1473-3099(15)00487-9. 26774243 PMC4792715

[44] Phuc BQ, Rasmussen C, Duong TT, Dong LT, Loi MA, Menard D, Tarning J, Bustos D, Ringwald P, Galappaththy GL, Thieu NQ 2017 Treatment failure of dihydroartemisinin/piperaquine for Plasmodium falciparum malaria, Vietnam. Emerg Infect Dis 23:715–717. doi:10.3201/eid2304.161872. 28322709 PMC5367417

[45] Imwong M, Suwannasin K, Kunasol C, Sutawong K, Mayxay M, Rekol H, Smithuis FM, Hlaing TM, Tun KM, van der Pluijm RW, Tripura R, Miotto O, Menard D, Dhorda M, Day NPJ, White NJ, Dondorp AM 2017 The spread of artemisinin-resistant Plasmodium falciparum in the Greater Mekong subregion: a molecular epidemiology observational study. Lancet Infect Dis 17:491–497. doi:10.1016/S1473-3099(17)30048-8. 28161569 PMC5406483

[46] White NJ, Pukrittayakamee S, Phyo AP, Rueangweerayut R, Nosten F, Jittamala P, Jeeyapant A, Jain JP, Lefevre G, Li R, Magnusson B, Diagana TT, Leong FJ 2014 Spiroindolone KAE609 for falciparum and vivax malaria. N Engl J Med 371:403–410. doi:10.1056/NEJMoa1315860. 25075833 PMC4143746

[47] Nguyen DV, Nguyen QP, Nguyen ND, Le TT, Nguyen TD, Dinh DN, Nguyen TX, Bui D, Chavchich M, Edstein MD 2009 Pharmacokinetics and ex vivo pharmacodynamic antimalarial activity of dihydroartemisinin-piperaquine in patients with uncomplicated falciparum malaria in Vietnam. Antimicrob Agents Chemother 53:3534–3537. doi:10.1128/AAC.01717-08. 19528277 PMC2715592

[48] McNamara CW, Lee MC, Lim CS, Lim SH, Roland J, Nagle A, Simon O, Yeung BK, Chatterjee AK, McCormack SL, Manary MJ, Zeeman AM, Dechering KJ, Kumar TR, Henrich PP, Gagaring K, Ibanez M, Kato N, Kuhen KL, Fischli C, Rottmann M, Plouffe DM, Bursulaya B, Meister S, Rameh L, Trappe J, Haasen D, Timmerman M, Sauerwein RW, Suwanarusk R, Russell B, Renia L, Nosten F, Tully DC, Kocken CH, Glynne RJ, Bodenreider C, Fidock DA, Diagana TT, Winzeler EA 2013 Targeting Plasmodium PI(4)K to eliminate malaria. Nature 504:248–253. doi:10.1038/nature12782. 24284631 PMC3940870

[49] Zou B, Nagle A, Chatterjee AK, Leong SY, Tan LJ, Sim WL, Mishra P, Guntapalli P, Tully DC, Lakshminarayana SB, Lim CS, Tan YC, Abas SN, Bodenreider C, Kuhen KL, Gagaring K, Borboa R, Chang J, Li C, Hollenbeck T, Tuntland T, Zeeman AM, Kocken CH, McNamara C, Kato N, Winzeler EA, Yeung BK, Diagana TT, Smith PW, Roland J 2014 Lead optimization of imidazopyrazines: a new class of antimalarial with activity on Plasmodium liver stages. ACS Med Chem Lett 5:947–950. doi:10.1021/ml500244m. 25147620 PMC4137381

[50] Chotivanich K, Tripura R, Das D, Yi P, Day NP, Pukrittayakamee S, Chuor CM, Socheat D, Dondorp AM, White NJ 2014 Laboratory detection of artemisinin-resistant Plasmodium falciparum. Antimicrob Agents Chemother 58:3157–3161. doi:10.1128/AAC.01924-13. 24663013 PMC4068498

[51] Dogovski C, Xie SC, Burgio G, Bridgford J, Mok S, McCaw JM, Chotivanich K, Kenny S, Gnadig N, Straimer J, Bozdech Z, Fidock DA, Simpson JA, Dondorp AM, Foote S, Klonis N, Tilley L 2015 Targeting the cell stress response of Plasmodium falciparum to overcome artemisinin resistance. PLoS Biol 13:e1002132. doi:10.1371/journal.pbio.1002132. 25901609 PMC4406523

[52] Lambros C, Vanderberg JP 1979 Synchronization of Plasmodium falciparum erythrocytic stages in culture. J Parasitol 65:418–420. doi:10.2307/3280287. 383936

[53] Chen LB 1988 Mitochondrial membrane potential in living cells. Annu Rev Cell Biol 4:155–181. doi:10.1146/annurev.cb.04.110188.001103. 3058159

