# Supplementary material for: Imidazolopiperazines Kill both Rings and Dormant Rings in Wild-Type and K13 Artemisinin-Resistant Plasmodium falciparum In Vitro
Source: Antimicrob Agents Chemother. 2018 Apr 26;62(5):e02235-17. doi: 10.1128/AAC.02235-17 (PMC5923180; doi:10.1128/AAC.02235-17)
Supplement: Supplemental material [file supp_62_5_e02235-17__index.html]

Supplemental material 

# Imidazolopiperazines Kill both Rings and Dormant Rings in Wild-Type and K13 Artemisinin-Resistant Plasmodium falciparum In Vitro

## Supplemental material

- Supplemental file 1 -

  Supplemental Figures S1 and S2

  PDF, 225K
