# Supplementary material for: Imidazolopiperazines Kill both Rings and Dormant Rings in Wild-Type and K13 Artemisinin-Resistant Plasmodium falciparum In Vitro
Source: Antimicrob Agents Chemother. 2018 Apr 26;62(5):e02235-17. doi: 10.1128/AAC.02235-17 (PMC5923180; doi:10.1128/AAC.02235-17)
Supplement: Supplemental material [file AAC.02235-17_zac005187150s1.pdf]

Imidazolopiperazines (IPZ) kill both rings and dormant rings in wild type and K13 artemisinin resistant *Plasmodium falciparum in vitro*

Laurent Dembele[1,2], Devendra Kumar Gupta[1], Michelle Yi-Xiu Lim[1], Xiaoman Ang[1], Jeremy J. Selva[1], Kesinee Chotivanich[3,4], Chea Nguon[5], Arjen M. Dondorp[3,6,7], Ghislain M.C. Bonamy[1], Thierry T. Diagana[1,8], Pablo Bifani[1,8,9§]

[1] Novartis Institute for Tropical Diseases, 10 Biopolis Road, #05-01 Chromos, Singapore, 138670

[2] Université des Sciences, des Techniques et des Technologies de Bamako (USTTB) ; MRTC – DEAP – Faculty of Pharmacy, Point G, P.O. Box: 1805, Bamako, Mali.

[3] Mahidol-Oxford Research Unit (MORU), Faculty of Tropical Medicine, Mahidol University, Bangkok, 10400, Thailand

[4] Department of Clinical Tropical Medicine. Faculty of Tropical Medicine, Mahidol University, Bangkok, 10400, Thailand

[5] National Center for Parasitology, Entomology and Malaria Control, Phnom Penh, Cambodia

[6] Oxford Centre for Tropical Medicine and Global Health, Nuffield Department of Clinical Medicine, University of Oxford, Oxford, UK

[7] Department of Intensive Care, Academic Medical Center, University of Amsterdam, Amsterdam, The Netherlands

[8] Department of Microbiology and Immunology, Yong Loo Lin School of Medicine, National University of Singapore, 119077 Singapore

[9] Singapore Immunology Network (SIgN), A*STAR, 8A Biomedical Grove, Immunos Building, Singapore 138648, Singapore.

[§]Correspondence: pablo_bifani@immunol.a-star.edu.sg

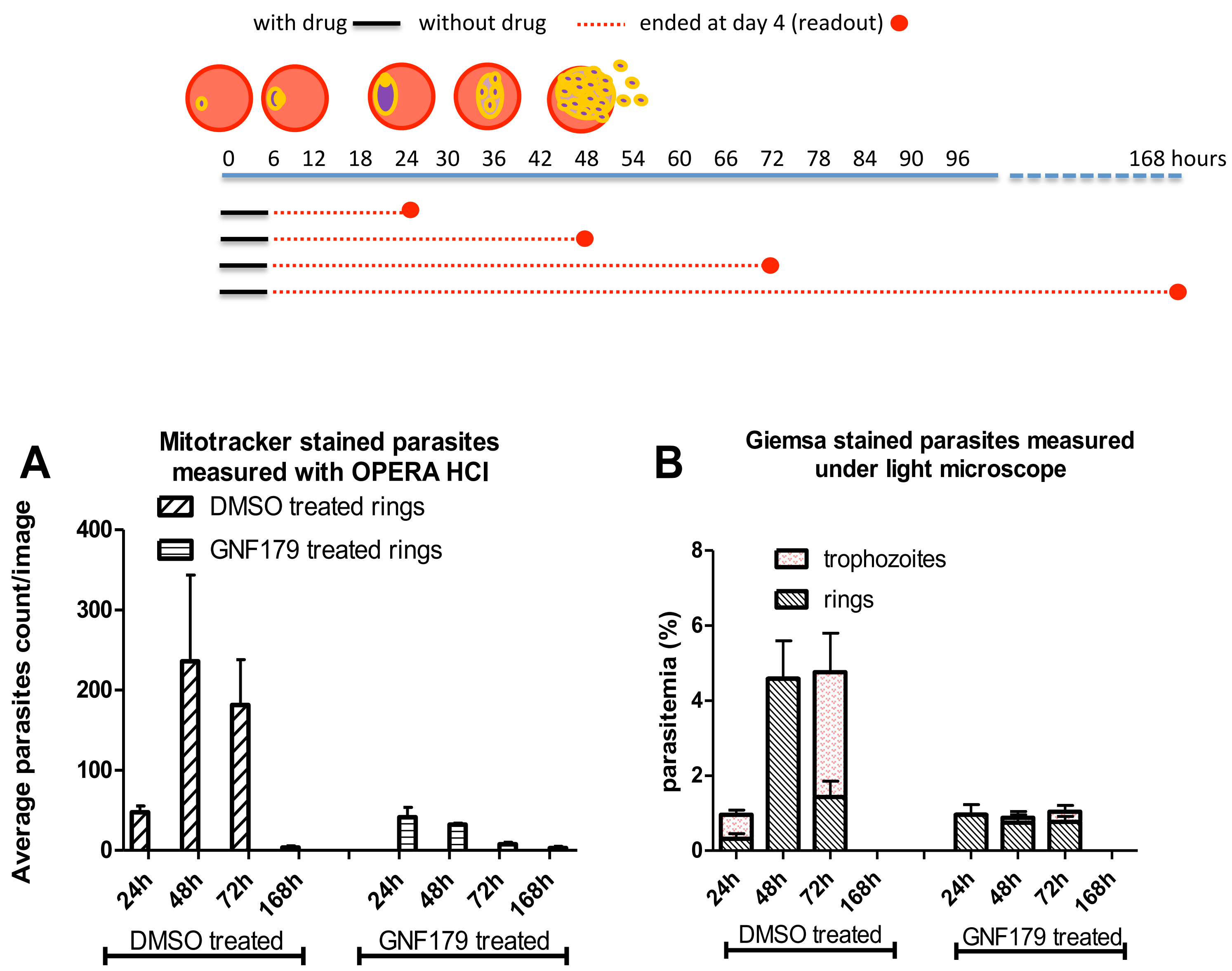

Supplementary figure 1. Killing kinetic quantification of GNF179 (100 nM), 6 and 24 hours exposure with Dd2 WT rings parasites (A) with MitoTracker® Orange stained live parasites counted using High Content Imaging (HCI); (B) with Giemsa stained parasites counted under microscope. Drug treatment was applied on 3-6 hours old ring stage parasites. Data are mean +/- SME and was done in three occasions.

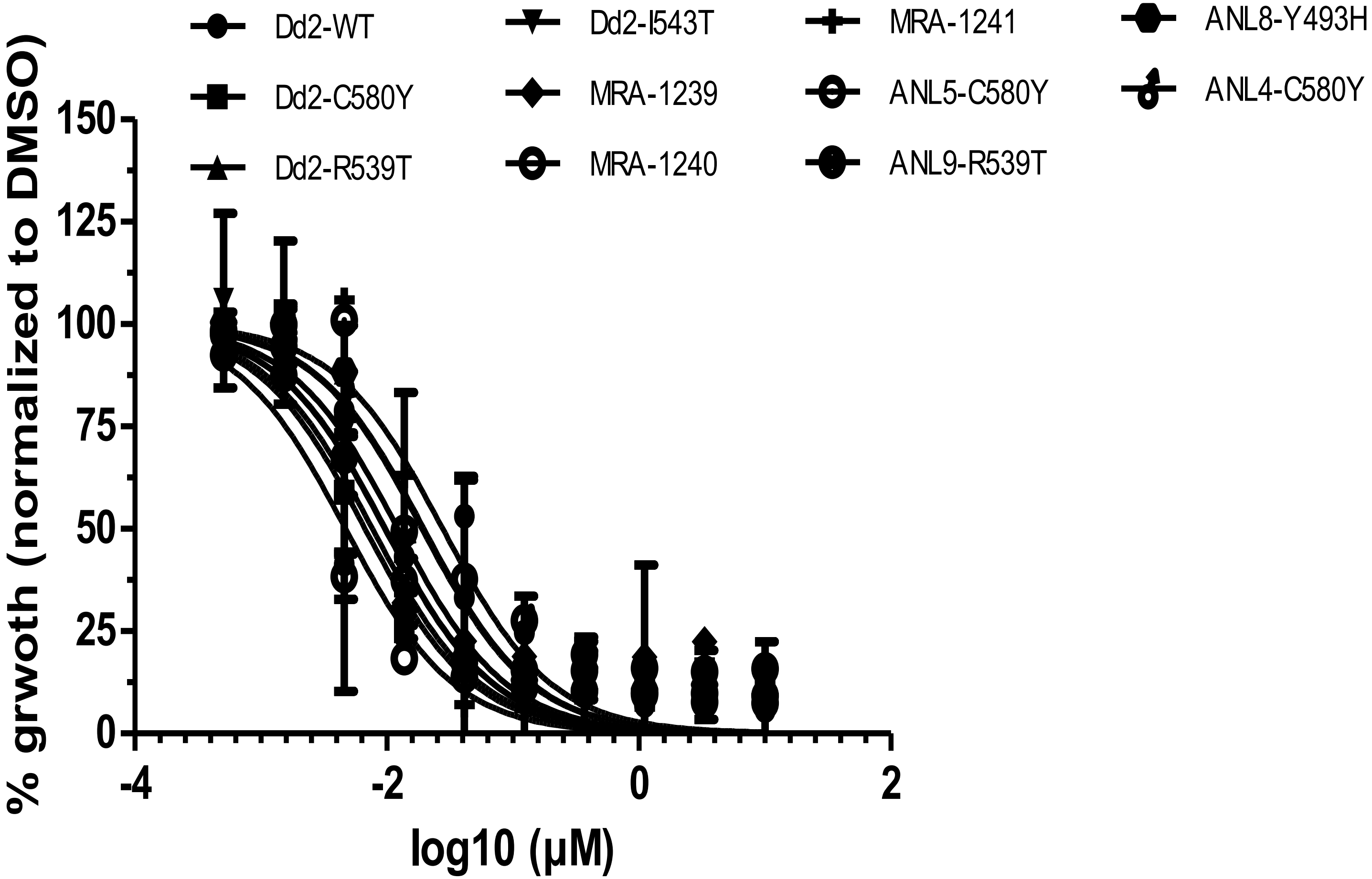

	Dd2-WT	Dd2-C580Y	Dd2-R539T	Dd2-I543T	MRA-1239	MRA-1240	MRA-1241	ANL5-C580Y	ANL9-R539T	ANL8-Y493H	ANL4-C580Y
IC50	0.006721	0.007700	0.01226	0.009644	0.009787	0.004707	0.01902	0.02782	0.009649	0.01226	0.01970

Supplementary figure 2. Dose response curves of GNF179 against transgenic K13 and WT Dd2 lines and ART resistant clinical isolates measured with 72 hours SYBR Green assay.
